# Applications of superparamagnetic iron oxide nanoparticles in drug and therapeutic delivery, and biotechnological advancements

**DOI:** 10.3762/bjnano.11.94

**Published:** 2020-07-27

**Authors:** Maria Suciu, Corina M Ionescu, Alexandra Ciorita, Septimiu C Tripon, Dragos Nica, Hani Al-Salami, Lucian Barbu-Tudoran

**Affiliations:** 1 Department of Molecular Biology and Biotechnology, Electron Microscopy Laboratory, Biology and Geology Faculty, Babes-Bolyai University, 5–7 Clinicilor Str., Cluj-Napoca, Cluj County, 400006, Romania; 2 Electron Microscopy Integrated Laboratory, National Institute for Research and Development of Isotopic and Molecular Technologies, 67-103 Donath Str., Cluj-Napoca, Cluj County, 400293, Romania; 3 Functional Sciences Department, Medical Faculty, University of Medicine and Pharmacy “Victor Babes”, 2 Eftimie Murgu, Timisoara, Timis County, 300041, Romania; 4 Biotechnology and Drug Development Research Laboratory, the School of Pharmacy and Biomedical Sciences, Curtin Health Innovation Research Institute, Curtin University, GPO Box U1987, Perth Western Australia 6845, Australia

**Keywords:** drug delivery, drug targeting, endocytosis, medical, nanoparticles, superparamagnetic iron oxide nanoparticles (SPIONs), toxicity

## Abstract

Superparamagnetic iron oxide nanoparticles (SPIONs) have unique properties with regard to biological and medical applications. SPIONs have been used in clinical settings although their safety of use remains unclear due to the great differences in their structure and in intra- and inter-patient absorption and response. This review addresses potential applications of SPIONs in vitro (formulations), ex vivo (in biological cells and tissues) and in vivo (preclinical animal models), as well as potential biomedical applications in the context of drug targeting, disease treatment and therapeutic efficacy, and safety studies.

## Introduction

**Figure 1:**
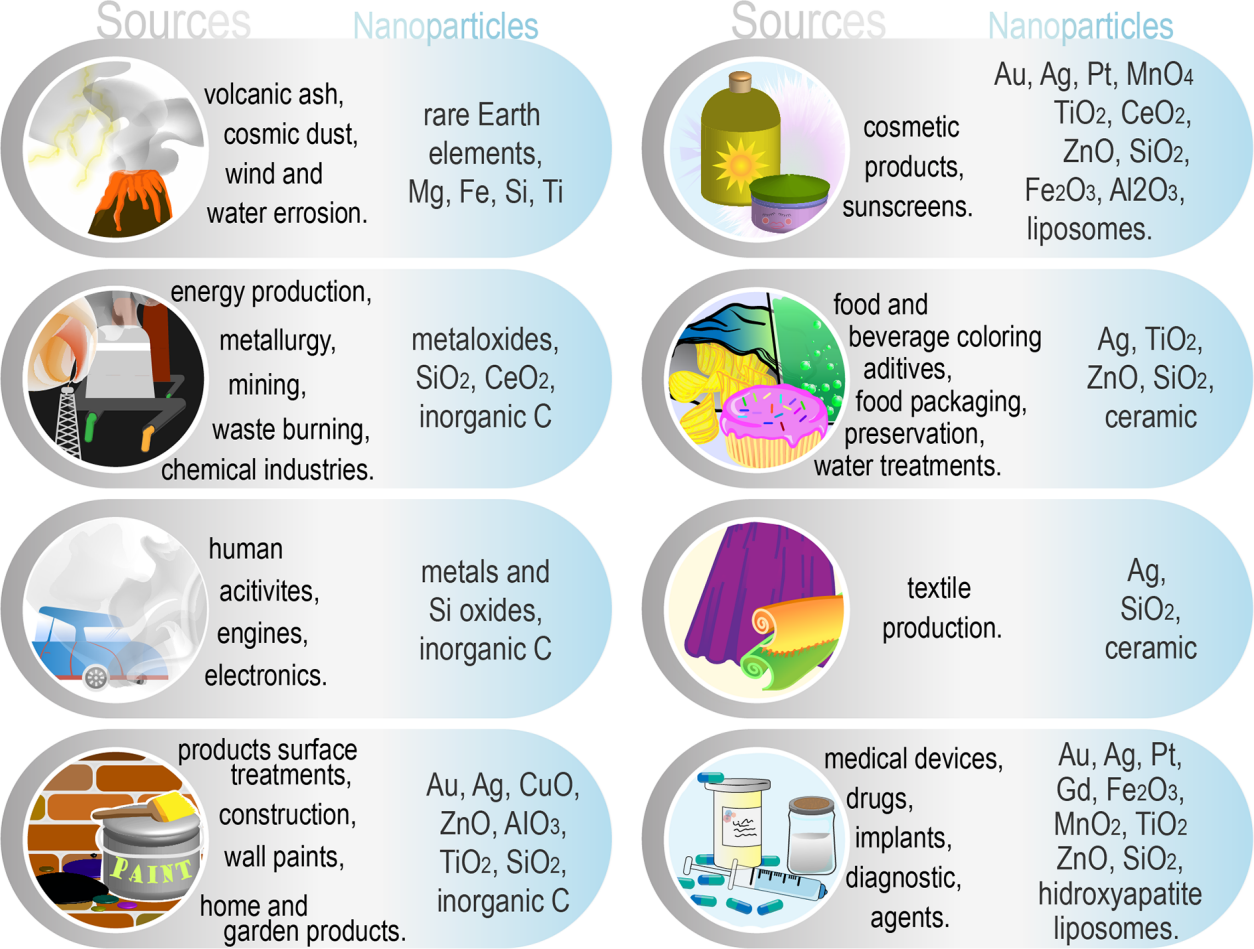
Common sources and types of nanoparticles that we come in contact with daily.

Nanoencapsulation technologies have been researched over the past several decades and have been widely anticipated to revolutionize current therapies [1]. The entire nanotechnology/nanomaterials branch is still very young but has rapidly expanded due to the many exploitable characteristics that nanomaterials have [2]. However, after decades of utilization, their effects are becoming transparent, and many of them reveal a very dark side [3]. We may not know it, but we come in contact with various types of micro- and nanoparticles every day (Figure 1), sometimes unwillingly, sometimes by choice [4–9].

As their name suggests, nanoparticles are particles of matter in the nanometer size range (10^−9^ to 10^−7^ m), one to three orders of magnitude smaller than the micrometer size range (10^−6^ m) and one order of magnitude above the atomic level (angstroms). They can be observed using specialized instruments only, such as electron microscopes or atomic force microscopes. At this level, the surface of the nanoparticles can become very large in relation to the volume, and the constituent atoms can be known in numbers. At the nanometer scale, the electromagnetic forces between the atoms gain importance and significantly influence the general behavior of the matter. This changes completely the physics and chemistry by which nanoparticles interact with each other or with other materials (didactically explained in a review by Roduner [10]). Also, a new branch of biology was formed, the study of nanoparticle interaction with biological structures, and the effects of nanoparticles on organisms and on the environment. Many nanoparticles showed great potential and proved their utility in biology and medicine. There are multiple types of nanoparticles routinely used in biology and related sciences for sensing, targeting or imaging, including quantum dots for fluorescence applications and electron microscopy (EM), iron oxide magnetic beads for the separation of cells and molecules, gold and silver nanoparticles as fiducials for EM, for immuno-EM labeling and surface-enhanced Raman spectroscopy, or for gene transfection, liposomes for drug delivery, and gadolinium or iron oxide nanoparticles for magnetic resonance imaging (MRI) (for more on this topic consult [11–14]).

Among the abovementioned nanoscience products, iron oxide nanoparticles, especially superparamagnetic iron oxide nanoparticles (SPIONs) hold a lot of promise in many domains, not only regarding biology [15]. SPIONs consist of iron oxides in the form of magnetite (Fe_3_O_4_) or maghemite (Fe_2_O_3_) and are easy to produce through a few well-documented synthesis methods yielding different forms and structures (e.g., round, cubic, hexagonal, clusters, core–shell with gold, silica, polymers, or surfactants). A lot of research is invested nowadays in developing coatings with certain characteristics, depending on the final application (e.g., biocompatibility/low toxicity, targeting a certain cell/compartment/molecule, or stability in biological fluids) [16]. SPIONs are superparamagnetic, which means that they are small enough to behave like one magnetic unit, rotating in the presence of a magnetic field without retaining the magnetism after the magnetic field is removed [17]. This property makes SPIONs good candidates for MRI, and also for a type of thermic treatment of cancer, called localized hyperthermia. There are also other types of nanoparticles used for MRI, but iron oxide is believed to be less harmful than other nanoparticles, such as lanthanides, which cannot be used on patients with kidney disorders [18], or manganese, which is neurotoxic [19]. Furthermore, excess iron oxides accumulate in liver and spleen as iron pool (ferritin and transferrin) for normal metabolic activities [20]. In localized hyperthermia, SPIONs generate heat by constantly aligning to an alternating magnetic field. This heat is rapidly transferred to the surrounding cancerous tissue in which proteins denature and, consequently, cells become apoptotic [21].

This review addresses the following points: There are SPION formulations commercially available for MRI for patients, but many studies still report toxic effects of the exact same type of SPIONs in vitro and in vivo. Of course, there are also many reports of positive aspects, with no downside to using SPIONs. Many of the specialists working in the development of SPIONs would say that it depends on the synthesis and the characteristics of the nanoparticles. However, some SPIONs previously approved for MRI where eventually redrawn from the market. So, which SPIONs are safe and which are toxic? Which studies should be trusted? With this review, we will try to shed some light on the aspects that make SPIONs toxic or biocompatible for medical applications. Although there are hundreds of studies on SPIONs that demonstrate their biomedical potential and applications, there still is no clear and definitive view of their safety hazards. The originality of this review is to highlight some of the inconsistencies within the publications regarding SPIONs, and to our knowledge this has not been published before.

For this review, we used the search engines Google Scholar, Google Books, Medline/Pubmed and Web of Science (WoS) for article and review papers and books reporting on effects, uses, in vivo and in vitro tests, toxicology, synthesis and properties of SPIONs. We used single terms or combinations of one or more of the listed terms. This search was carried out for the period between June 2015 and January 2020. The graphics of publication counts per year were obtained using the values obtained from Web of Science Core Collection database and Medline (PubMed) trend website [22–23], using the following keywords: "superparamagnetic iron oxide", "SPION", "iron oxide medical" for the period of 2000–2019.

## Review

### Benefits of SPIONs or what makes SPIONs such a promising aspect for therapeutics and adjunct treatments

Firstly, certain SPIONs are already clinically approved for magnetic resonance imaging (MRI) [24–26]. For this application, SPIONs have been functionalized with dextran, dendrimers, albumin, silicones, liposomes, poloxamer, poly-ʟ-lysine, sugars, or polyethylene glycol (PEG) [27–33]. Hyperthermia treatment for cancer therapy is still under scrutiny. It shows great potential due to the property of SPIONs to produce local heat when placed under an alternating magnetic field [30]. Currently, it is used only as an alternative therapy and nearly always in combination with other therapies [34]. Results have shown that even non-magnetic hyperthermia (water-bath method) using SPIONs has cytotoxic effects [35]. Many studies focus on the potential of SPIONs as contrast agents and/or carriers of fluorescent markers [35–37] and on the fact that SPIONs can be guided to target sites in the body by means of an external magnetic field or receptor targeting [33,38–39]. Other potential uses for SPIONs are non-viral gene therapy and cell selection [15,40–42], in vivo cell tracking [43–44], in vivo labeling using iron isotopes [45], tissue repair [46–48], and tissue enhancement [49].

The principal property that makes SPIONs very attractive is that the same type of nanoparticles is produced and stored naturally by biomineralization and can be found in many species including humans. There are reports of natural magnetite and ferritin formation in the brain and in tumors [50]. When degraded in the body (but only up to certain concentrations/doses), SPIONs are turned into nontoxic iron ions, and are stored in the liver [38]. If the concentration is too high, this conversion does not occur anymore and the SPIONs become highly toxic. Also, it is important to keep in mind that there are tumors that are stimulated into mutagenesis and malign status by an increased iron concentration. Therefore, many possible treatments via iron chelation are in clinical trials [51]. A recent report suggests that submandibular gland cells suffer epigenetic mutations when treated with maghemite [52]. This fact becomes very important especially when developing SPION treatments for cancer. Unfortunately, to date scientists and physicians cannot provide definite protocols and dosages for the safe usage of SPIONs [26].

SPIONs have immense potential in diagnostics and treatment of various diseases, and also, in biotechnology and research. However, there is still a lack of understanding about how to synthesize SPIONs that are truly safe for human application and also for the environment.

### Why is it so difficult to assess the safety of SPIONs?

**Figure 2:**
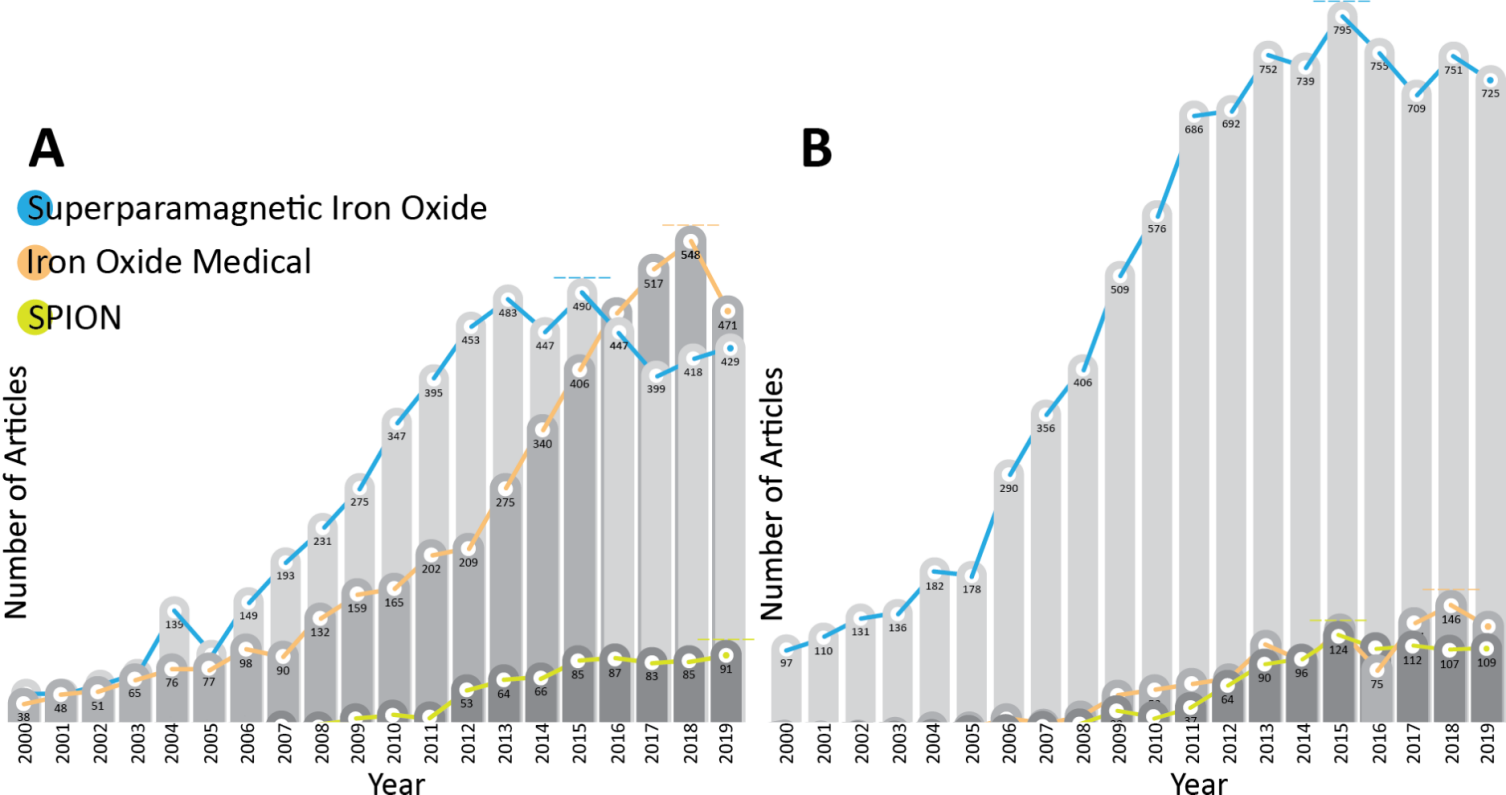
Number of publications per year on the topic of superparamagnetic iron oxide with biological and medical applications between 2000 and 2019. (A) Values obtained using the Web of Science database (total counts per search – blue: 9575, orange: 1099, green: 966). (B) Values obtained using Medline (PubMed) trendline count (total counts per search – blue: 5610, orange: 4436, green: 744).

There are literary thousands of papers that analyze the effects of SPIONs in biological settings (11640 according to WoS and 10790 according to Medline search, in the period between 2000 and 2019; Figure 2). Each paper brings new information, but the big picture is still unclear. To give the reader an idea we will shortly describe some of the reports involving SPIONs which consider similar problems. Studies concerning the synthesis of SPIONs is presented in the next section, followed by sections on the physical, chemical and biological properties of SPIONs.

Various aspects regarding the synthesis of SPIONs and the application to cell biology or medicine have to be considered. Some properties of SPIONs can be controlled through synthesis, such as core type, oxidation state, functionalization, dimension, form, and concentration, and some of the properties result from the primary properties mentioned above (zeta potential, secondary conjugation and interaction with serum and cells components, hydrodynamic dimension, dispersibility, and magnetization). However, a few very important properties are still not completely understood and cannot be controlled yet (cell contact breakdown products, secondary conjugation and corona formation, and the interaction with cells and blood components) [27,46,53–54].

Many papers state that magnetic iron oxide particles left “naked” or unconjugated exhibit highly negatively charged and hydrophobic surfaces that induce aggregation and/or the formation of large clusters. When in contact with biological structures, this mechanism provokes capillary clotting and reduces tissue and cellular absorption. To prevent this, nanoparticles are coated with stabilizers, which are added during preparation [30], but the coating material can be damaging to the sensitive balance of the cell metabolism, if not even toxic [55]. Also, iron is believed to be responsible for liver cancer (iron-induced carcinogenesis) and some cell mechanisms seem to support this possibility (including the generation of reactive oxygen species, ROS, which can potentiate direct damage to DNA and proteins, and induce lipid peroxidation) [24,56]. It was also shown that histidine–proline-rich glycoproteins with high molecular weight, e.g., kininogen and plasma prekallikrein, from blood serum attach strongly to the surface of negatively charged SPIONs [57], together with other weak binding proteins, i.e., mannose-binding lectin and their associated serine proteases, apolipoproteins, beta-2 glycoprotein, and clotting factors FXI and FXII. SPIONs with no surface charge have no proteins adsorbed onto them [58–59]. These characteristics, properties, and effects were observed in numerous studies made with SPIONs on animal and human cells in vitro and in vivo, and are concluded at the end of the review. However, these observations are often contradictory.

### Iron oxide compounds and geometric shapes

Iron oxides can be found as magnetite (Fe_3_O_4_), maghemite (γ-Fe_2_O_3_) or hematite (α-Fe_2_O_3_) [60]. There are various geometric forms of SPIONs, which depend on the synthesis. The most extensively studied form are spherical SPIONs, followed by cubic, hexagonal, rod-like, octagonal, nanoworm, and octopod (star-shaped) SPIONs [61–62]. Non-spherical SPIONs are getting increasingly more attention because of their improved efficacy in various domains. Single-domain cubic iron oxide particles (20 nm) are performing better in hyperthermia applications than similar spherical particles. Also, for hyperthermia purposes pure iron cubic nanoparticles are considered the most effective, but unfortunately pure iron is toxic because it leads to high oxidative stress. To avoid this problem there is a lot of ongoing work regarding the design of core–shell particles with pure iron cores [63–64]. Octopod SPIONs (30 nm) were also shown to be better than spherical ones for MRI, having increased contrast and no toxicity in vitro and in vivo [61]. Iron oxide nanoworms were found to be a better solution for targeting tumors (in vitro) and for accumulation in tumors (in vivo) than simple spherical SPIONs [62].

### Dimensions: synthesis dimension and hydrodynamic dimension

The dimension of a nanoparticle has two values, i.e., the synthesis dimension and the real dimension in dispersion media. The physical dimension of the nanoparticle obtained at the end of the synthesis process is measured with an electron microscope and is important for the physical and chemical properties of the nanoparticle. The real dimension of a nanoparticle is the one measured in suspension in the colloidal state. It differs depending on suspension medium, pH value, concentration, and ionic strength. The hydrodynamic dimension is much larger than the real dimension of a nanoparticle, as it also measures the layer of solvent and solutes permanently or transiently retained on the nanoparticle surface. For example, ultrasmall SPIONs of 10 nm or less (synthesis dimension) can measure a hydrodynamic diameter of 300 nm in water when uncoated, and around 100–150 nm when functionalized. If the same nanoparticles are measured in a culture medium, phosphate buffer, or blood serum, the hydrodynamic diameter can vary from hundreds of nanometers to few micrometers, due to corona formation or aggregation [54]. Therefore, the hydrodynamic diameter is important for biological and medical applications [65–66].

Some authors state that SPIONs having sizes between 5 and 10 nm are best for certain slow drug release treatments [46], however, others report that these ultrasmall SPIONs are dangerous due to their large in-tissue dispersity [67]. Other reports suggest that very small (less than 10 nm) and very large (more than 200 nm) SPIONs are to be considered dangerous for the human organism, and that the intermediate range from 30 to 50 nm should be used for nanomedicine [68]. Very small nanoparticles can easily enter a cell nucleus inducing DNA damage [69], and some authors emphasize that ultrasmall SPIONs can be used for gene therapy because of that [70]. SPIONs with diameters of about 100 nm are favored for their good surface-to-volume ratio and dispersion properties [71]. Also, the smaller the nanoparticle is, the longer it is retained in the blood circulation [72], which could also lead to capillary blockage. Despite these issues, 5 to 15 nm SPIONs are currently approved and used for human medical applications, such as MRI, even with acknowledged side effects [73–74]. These features are very important in in vivo applications because different types of cells internalize nanoparticles only at certain dimensions, as shown in in vitro tests. Larger particles, exceeding 500 nm are endocytosed to a far smaller extent [75–76].

### Surface coating, charge, and corona

Due to the fact that iron oxides have a certain toxicity when in direct contact with cell components, SPIONs designed for biological applications are synthesized with biocompatible coatings. This coating is very important as its chemical composition determines the way a cell responds to the nanoparticle contact. The coating may or may not have a surface charge that faces the surrounding media, and molecular components from the media may be drawn to the nanoparticle surface to form layers of adsorbed molecules. These layers are called corona and may be important for nanoparticle recognition by the reticuloendothelial system and for endocytic pathways [77].

Naked iron oxide nanoparticles were repeatedly shown to be toxic in vitro and in vivo [24]. Also, the synthesis of nanoparticles larger than 20 nm requires a certain surface coverage of the nanoparticles to obtain a monodisperse colloidal solution and to prevent the aggregation of nanoparticles. The substances used for the synthesis are either lipids or surfactants. In contact with a living cell, the lipids will be stripped from the particle, leaving the bare nanoparticle in direct contact with the biological material, thus, inducing cytotoxicity. Some surfactants may not be biocompatible because they can disturb the lipid and protein metabolism [78]. In order to use SPIONs for medical applications it is necessary to cover the SPIONs with a biocompatible material that prevents the aggregation of the nanoparticles. Nanoparticle coating or conjugation has the advantage of creating a designed surface chemistry for specific applications. This coating has a downside as well. It reduces the hyperthermia capabilities of nanoparticles because it lowers the magnetic saturation and it can also alter the stability of the nanoparticles [79]. For drug delivery the surface functionalization is critical. Studies have shown that covalent binding of drugs can enable slow drug release at the targeted site. A functionalization that permits only adsorption can lead to premature drug release [33,46,80].

Mojica Pisciotti and co-workers [39] studied the effects of dextran and PEG coatings on two animal kidney cell lines and showed that dextran-coated SPIONs are not cytotoxic even at a concentration of 400 µg Fe/mL, in contrast to the PEG-coated SPIONs, which reached 50% toxicity at 100 µg Fe/mL. Dextran was shown to get stripped from the nanoparticles in macrophages that were later apoptotic. The authors concluded that apoptosis was caused first by dextran intoxication and later by iron oxidative stress [81]. Dextran-coated SPIONs were found to accumulate in large amounts in tumor sites in mice, in contrast to PEG-coated SPIONs, which did not accumulate, even in the presence of an external magnet at the tumor site. The PEG-coated SPIONs exhibited a longer blood circulation time than dextran-coated SPIONs [39], having better colloidal stability and reduced agglomeration tendency. In addition, very importantly, the PEG coating did not degrade the magnetization properties of the nanoparticles [82]. Chen et al. [40] showed that SPIONs functionalized with PEG, grafted with polyethyleneimine and conjugated with CD44 siRNA can be used as non-viral gene therapy vectors. Polyethylene imine is the material of choice for nucleic acid intracellular delivery, as it gives a positive charge to the nanoparticle surface [41].

Polyvinyl alcohol (PVA)-coated SPIONs were tested as a drug delivery system in sheep joints. The authors found that PVA-coated SPIONs in the presence of an external magnet accumulated at the joint and remained there until at least the fifth day of treatment [83]. Many coatings have been tested for biomedical applications. PVA is one of the most commonly used for SPION coating, yielding good nanoparticle stability and no cytotoxicity. It is currently the preferred coating for MRI nanoparticles [54,76,84].

Depending on the type of emulsifier used, the coating can be hydrophilic or hydrophobic, but it is common to use hydrophilic polymers, such as PVA or PEG, for drug release studies [33,76] because the hydrophobic polymers are rapidly uptaken by macrophages [85]. SPIONs coated with a mixture of PVA and polyvinyl amine were shown to induce very active mitochondrial and endocytic processes in cells and to be highly toxic to cells due to the positive charge of the amine groups. The uptake was determined to be proportional to the number of amine groups [84]. If the amine groups were exchanged with carboxyl or thiol groups, the activity was reduced and cells did not internalize as many nanoparticles [86]. In vivo tests also showed that PVA-coated SPIONs are biocompatible, but a higher number of amines in the coating is not compatible with the animal metabolism [84]. Even so, Strehl et al. [26] concluded in their paper that PVA-coated SPIONs with amines had no toxic effect on immune cells. Instead, they actually determined a viability increase in macrophages. The general consideration is that polymer coating offers colloidal stability, but in fact PVA-coated SPIONs are only stable in water at a certain pH value. In cell culture medium they agglomerate. Studies showed that the components of the medium, and not the calf serum added to the medium, led to nanoparticle agglomeration. Dulbecco's Modified Eagle Medium (DMEM) with added serum yielded the highest nanoparticle stability compared to other media [54,87]. Also, PVA-coated SPIONs were not internalized by cells when the cell culture medium had serum in it. Without serum cell uptake occurred [84].

Polyvinylpyrrolidone (PVP)-coated SPIONs showed an unexpected effect on human breast cancer cells (BT-474) in vitro. At concentrations between 10 and 100 µg/mL, for up to three days, nanoparticles doubled the metabolic activity and the population number of cancer cells. Only at concentrations higher than 200 µg/mL did the nanoparticles become toxic to these cells. This effect was noticed only when using PVP coating and only on BT-474 cells, and the authors confirmed this by testing with dextran-coated SPIONs [88]. These results point out the fact that not all cells respond in the same way to a certain type of coating. This becomes very important when nanoparticles are administered in vivo, as we cannot always determine exactly the nanoparticle concentration at the target site of the nanoparticles.

SPIONs coated with polyglycerol and folic acid were shown to have no toxic effect on HeLa cells up to 400 µg/mL, but the coating enhanced the effect of the hyperthermia water-bath treatment [45]. This effect of biocompatibility at 37 °C and cytotoxicity at 42 °C, even at micromolar concentrations, was noted already earlier by other groups [89–90]. Recently, in a study of SPION stability in cell culture media, it was demonstrated that poly(methacrylic acid)-coated SPIONs and citric acid-coated SPIONs are stable in common cell culture media (DMEM, RPMI), when serum was added, but produced aggregates larger than 1 µm in simple media and in phosphate buffer [54].

Stability and interaction with cells depend on surface charge and zeta potential. A higher zeta potential (positive or negative) leads to more stable and dispersed particles in the colloid [76,91]. Positively charged particles tend to stick to the cell surfaces (which have a negatively charged outer layer in general) and were reported to be toxic in utero [92]. Highly negatively charged SPIONs were reported to be slightly toxic in vitro and in vivo. They are easily accumulated and degraded by the liver Kupffer cells and parenchymal cells [92–94]. The zeta potential and the ionic character of particles as well as the corresponding interactions with cell membranes can be changed with different conjugation ligands (type, proportion, and concentration). Cardoso et al. obtained 10–15 nm SPIONs covered with dopamine, tiron, glycerol, and *o*-phosphorylethanolamine in different combinations and proportions, which had zeta potential values ranging from −40 to +20 mV and formed stable colloids. The zeta potential and the ligands present at the SPION surface showed equal importance in determining the amount of iron oxide engulfed by the cells [95]. Hydrophobic particles are rapidly recognized and engulfed by the reticuloendothelial system [85,96]. Hennig et al. [97] obtained lauric acid/BSA-coated and dextran-coated SPIONs with neutral surface charge, which remained colloidally stable without forming any aggregates. This feature is very important for cell endocytosis, as aggregates precipitate between cells in vitro and therefore give incorrect results. Stable and well dispersed nanoparticles are internalized to a higher extent [54].

**Figure 3:**
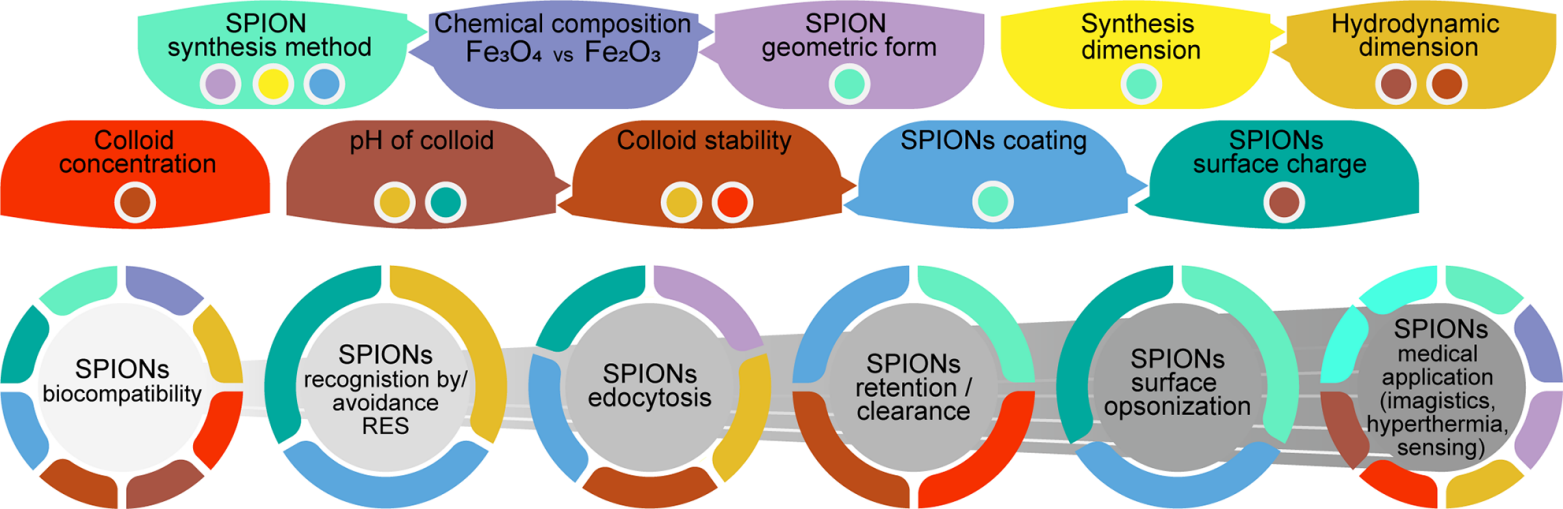
Schematic representation of how the physical and chemical characteristics of SPIONs influence biological response and biomedical applications.

A lot of effort has been spent in this area and today there are methods to obtain very stable SPIONs functionalized with PVA, PEG, dextran, chitosan, and poloxamers, which can be used in biological fluids at physiological pH values and which remain stable with low toxicity in vitro [38,46,98–103]. Still, Hong and collaborators [104] stress the fact that we should pay more attention to the charge effects of SPIONs because not all types of cytotoxicity can be easily and readily detected. Figure 3 gives a schematic overview of how the physical and chemical characteristics of SPIONs influence biological response and biomedical applications.

### Endocytosis, exocytosis, retention and clearance

There are a number of possible cell entry mechanisms for nanoparticles, such as clathrin and/or caveolae pits, RhoA, CDC42, ARF6, or ﬂotillin-mediated endocytosis/phagocytosis and receptor-independent macro- and micropinocytosis (for reviews on the endocytosis of nanoparticles see [105–106]). Macropinocytosis is not selective and can take up spheres with a diameter of up to 1 mm [81,107]. The exact mechanisms are not yet known or understood, and many studies regarding the endocytosis of SPIONs are still underway [108]. It is believed that size is the major criterion regarding endocytosis [27], and this was also noticed by our group [109]. Exocytosis has been less extensively studied in vitro and is cytoskeleton-mediated. It has been noticed and reported as nanoparticle recycling [110]. A study found that the uptake of SPIONs by cells in vitro is depends on the cell cycle phase. SPIONs are not exocytosed by dividing cells. Instead, they are split between daughter cells when the cell divides [111]. Another study compared the endocytosis of gold and iron oxide nanoparticles in a co-exposure experiment. The authors found that the cells are not selective and internalize nanoparticles of both types. However, they found that the co-exposure induces more endocytosis events than the single exposure to only gold or iron [112]. Cell endocytosis, retention, and exocytosis become very important when nanoparticles are used for various types of treatments. There are reports of a reduced hyperthermia effect when nanoparticles are located in endosomes, and reports of spontaneous endosomal escape [113–114]. For the specific treatment of cisplatin-resistant nasopharyngeal carcinoma, Weng et al. [115] produced SPIONs covered with cisplatin and with TAT peptide (a HIV-derived cell penetrating peptide). Their nanoparticles had an increased endocytosis rate, which, combined with the Fenton reaction of the iron oxide core taking place in the lysosome and the cell cytoplasm, increased the cancer cell response to cisplatin and reversed the resistance of the cells to the drug [115].

The retention time of nanoparticles in tissues and the clearance from blood and/or specific organs is very important for medical applications. These properties depend on the methods used, targeted organs/tissues, image resolution, administration way and dosage, and toxicity [116]. Fakhar-e-Alam and collaborators [117] observed that above a certain dose cancer cells develop a resistance to ligand-dependent endocytosis.

Opsonized dextran-coated SPIONs between 1.2 and 4.6 nm are taken up by macrophages through receptor-mediated endocytosis and not pinocytosis, followed by degradation in lysosomes [118]. In a study comparing dimension, concentration, and time of action on macrophages of SPIONs, Huang et al. [27] concluded that intermediate SPION dimensions (around 37 nm) are the most effective for uptake. Also, higher concentrations led to a higher uptake (up to 250 μg/mL). The authors also found that the maximal uptake was registered 4 h after the treatment with SPIONs and the declining plateau was established 6–8 h after treatment [27]. Kasten et al. [30] found that for a higher concentration of nanoparticles and a more positive zeta potential the endocytosis rate is increased.

A few papers have reported on the circulation times of nanoparticles in blood. Some state that SPIONs with a diameter below 20 nm seem to have a prolonged circulation time [72,119]. This is also the case for SPIONs with cross-linked PEG on the surface [120]. Others showed that large commercial SPIONs with dextran coating (200 nm) and very small SPIONs (below 10 nm) have a limited circulation time because they are very quickly recognized by macrophages and transported to liver and spleen [121] or eliminated through the kidneys [68]. Alternatively, they can be accumulated in lymph nodes, thus having a longer circulation time [36,122]. The general opinion is that smaller SPIONs are opsonized to a lesser extent, which leads to a lower rate of recognition by macrophages and monocytes. Also, a highly positive or negative surface charge of nanoparticles leads to a very small blood circulation time of only a few minutes [36]. Bare SPIONs are also rapidly cleared from blood after intravenous injection. They are transported to liver, spleen, and lymph nodes and engulfed by the reticuloendothelial system [123]. SPIONs reach lymph nodes either directly through endothelium and macrophage phagocytosis, or through non-selective endothelial transcytosis into the interstitial space and draining by lymphatic vessels [36]. This problem limits the usefulness of SPIONs as targeting agents [124]. Macrophage recognition of nanoparticles can be used for the desired treatment as well. Ullah et al. used the rapid macrophage endocytosis of silica-coated SPIONs loaded with maytansin, an antiproliferative toxin. They infiltrated these macrophages in spheroids of tumor cells and destroyed the cells by hyperthermia in vitro [125]. Moghimi et al. [124] found that liposomes and polymeric nanospheres used as nanocarriers are also opsonized, which promotes their clearance by macrophage activation. The opsonization also leads to toxicity by a complementary activation of downstream pathways and this could lead to unwanted side effects.

Simberg et al. [58] studied the impact of SPION opsonization on the blood retention. They identified an unexpectedly large number of proteins on the surface of dextran-coated SPIONs, including histidine–proline-rich glycoprotein, kininogen, prekallikrein, mannose-binding lectins, complement factors, clotting factors, immunoglobulins, fibronectin, and fibrinogen. They obtained the equivalent of 1 mg of proteins bound to 1 mg of iron oxide (in the case of extensive washing). This corona of proteins does not completely cover the nanoparticles. Their results indicated that opsonization has no influence on macrophage recognition [58], and that the activation of the kinin–kallikrein system, which influences inflammation, blood pressure, coagulation, and pain, has no influence on tumor microvascular clotting [126]. The authors suggest that macrophage recognition might be caused by the incomplete coverage of dextran on the iron core [58].

Qiao et al. [36] found that SPIONs coated with PEG have a longer blood retention time by evading the reticuloendothelial system. Also, they tend to accumulate at tumor sites making them good candidates for MRI imaging. 300 nm SPIONs coated with dextran were cleared from the main accumulation sites (liver, spleen, lungs) after 72 h, but the same SPIONs covered with silicon were still accumulating after 72 h. Similar results were obtained using 10 nm polyethyleneimine-coated SPIONs, which accumulated only in liver and spleen, with a maximum at 24 h [41]. Jain et al. [127] observed that 55% of the administered SPIONs were found in the liver after 6 h. They were gradually transformed to ferritin and hemosiderin, and then transferred to the spleen where they were kept for storage similar to the Fe collected from dead red blood cells. This can only happen up to the maximum capacity of the spleen [67].

Studying the diffusion of SPIONs in the brain for MRI, Wang and collaborators [128] showed that dextran-coated SPIONs (20 nm) have a good dispersion in the interstitial space of the rat brain, with a retention maximum at 6 h after injection and a clearance of two weeks. When using gold-coated SPIONs they noticed that all nanoparticles remained close to the injection site and were internalized after 24 h and resistant to clearance. Kasten and et al. [30] found that starch-coated SPIONs (BNF-starch) were taken up by human adipose tissue-derived stem cells in a dose-dependent manner. After 24 h these SPIONs ended up in lysosomes where they were degraded to Fe ions. Arbab and collaborators [129] attempted to find out the necessary lysosomal pH value for the iron metabolism to complete. They found that mesenchymal stem cells needed a sodium acetate buffer with pH 4.5 to completely reduce the iron, which took them less than seven days. At pH 5.5, they obtained a measurable iron release after 48 h. To prevent the lysosomal degradation of SPIONs, Wu et al. developed a method of including uncoated SPIONs in hollow carbon spheres. They showed that the nanoparticles remained unaffected by cellular degradation and maintained their magnetization properties [130].

Hennig et al. studied the way nanoparticles cross human arterial walls under real flow conditions when under magnetic influence. They used two types of SPIONs, namely lauric acid/BSA-coated and dextran-coated SPIONs, and observed that when used in plasma or in whole blood, they produce clotting. However, both types of SPIONs cross the walls and accumulate in the tissue [97]. Wei et al. used 80 nm PEG-coated SPIONs capped with epidermal growth factor and green fluorescent protein to target atherosclerotic plaques for MRI detection in vivo with good biocompatibility and good targeting resolution, showing that for this application retention time and dimension of the PEG-coated SPIONs were ideal [131].

Regarding commercial SPIONs, reports indicate that Ferumoxide/Endorem can be found in chondrocytes for up to 12 weeks, at which time they still yield a good MRI signal [132]. Feraheme was detected for 40 days in myocytes in vivo [133].

### SPIONs applications: hyperthermia, imagistics, detection

SPIONs have been extensively examined regarding biocompatibility, targeted or intended cytotoxicity (ferroptosis), local hyperthermia treatments, photothermal or photodynamic therapy, and MRI. Also, there are increasingly more studies reporting on combinations with in vivo fluorescence imaging, sensing and detection. SPIONs have gained a lot of interest in theranostics, which combines diagnostics and treatment by a single intervention, where they show a lot of promise [64,134–138].

Depending on the dimension of the SPIONs, the heating during hyperthermia is determined by Néel relaxation for SPIONs smaller than 10 nm, by Néel and Brownian relaxation for SPIONs between 10 and 13 nm (mechanisms in superparamagnetic nanoparticles), and by hysteresis loss for larger SPIONs (mechanism of ferromagnetic nanoparticles) [139].

Hyperthermia potential and efficacy of SPIONs depend on their structure, size, volume, magnetic anisotropy, concentration, and the potential to remain dispersed and not to agglomerate at the site of action [63]. Hyperthermia only works if the nanoparticles have a single magnetic domain, i.e., if they behave uniformly throughout the entire mass as a single magnet. There are studies referring to the dimensions of single domains for iron oxides. Up to 25 nm magnetite exhibits superparamagnetic behavior and around 80 nm it exhibits ferromagnetic behavior. Maghemite is superparamagnetic up to 35 nm and ferromagnetic up to 90 nm [140]. The ideal nanoparticle for hyperthermia has a high surface absorption rate, which means that a small amount of particles releases a large amount of heat in a short magnetization time window. It is not yet established if size is the decisive factor in hyperthermia treatment, as different authors offer contradictory reports [141]. Our studies also proved that different cells respond to hyperthermia treatment at different time scales [142]. However, there are in vivo hyperthermia experiments that showed that the best size range for SPION is between 15 and 50 nm, and that cubic nanoparticles have the highest surface absorption rate [143]. The downside of cubic nanoparticles is that they have a higher tendency to aggregate at high concentrations than spherical SPIONs, which eventually affects the hyperthermia efficacy. Despite this, cubic SPIONs are more effective in hyperthermia [63]. This was proved in vitro [144] reaching local temperatures of 48 °C (after 1 h treatment at 15 mT and 150 kHz) and effectively killing breast cancer cells [145]. A high surface absorption rate can be obtained by increasing the frequency of the magnetic field, but that can only be done in a biologically safe limit, determined by Brezovich and Atkinson [146–147] as *H*·*f* ≤ *C*, with *C* = 5·10^9^ A·m^−1^·s^−1^. A study from 2007 showed that the best conditions regarding safety and hyperthermia effect can be obtained at *f* = 400 kHz [148]. A significant increase of temperature might not be required for killing cancer cells, as Creixell and collaborators showed in 2011. They did not obtain a measurable difference in temperature while still more that 70% of the cells died by apoptosis after hyperthermia treatment [149].

There are nanoparticle combinations of iron with zinc, manganese or nickel that have better heating properties under an alternating magnetic field. They were, however, shown to be highly toxic and inappropriate for medical applications [150–151]. Most studies have demonstrated that covering SPIONs with non-magnetic compounds can dramatically degrade their magnetization properties [38]. Even so, Alhasan et al. synthesized 9 nm SPIONs covered with PEG and adsorbed on reduced graphene oxide sheets, which enhanced the thermal effect of hyperthermia and reduced the viability of breast cancer cells to less than 25% by reaching 43 °C [152]. Also, Zuvin et al. used 4–5 nm SPIONs conjugated with poly(acrylic acid) and anti-HER2 antibody against breast cancer cells and obtained a temperature of 41 °C in just 2 min of magnetization at 400 kHz, 1 mT and 0.8 kA·m^−1^, which killed only the cells that had engulfed nanoparticles due to the low magnetization and high thermal efficacy of the SPIONs [134]. Studies show that hyperthermia increases the efficacy of standard cancer treatments. When used in combination with chemotherapy, radiotherapy or radiofrequency ablation, or photothermal therapy, the final outcome of the combined therapy would be more beneficial to the patient, although in clinical practice, physicians are reluctant to use these methods [136,153].

Recently, SPIONs were observed to be good candidates for photothermal and photodynamic therapy, using near-infrared (700–2000 nm) laser excitation of the nanomaterial. For these therapies, SPIONs are theoretically preferred in larger clusters, although studies have shown that they can yield up to 12 °C temperature increase even in the ultrasmall range (4–5 nm SPIONs at 785 nm laser wavelength, 800 mW power for 20 min) [136]. The study concluded that ultrasmall SPIONs can also produce heat by excitation with wavelengths smaller than near infrared and that the heating efficacy depends on the laser power.

Some papers even report a better heating efficacy for SPIONs formulated as magnetosomes used in photothermal therapy than for magnetized cubic SPIONs. The authors demonstrated that magnetization hyperthermia is up to two orders of magnitude less effective in cells and tissues than in water. In contrast, photothermal therapy is not influenced by tissues leading, therefore, to an up to 1000 times better heating efficacy than magnetization, but only at significant concentrations of at least 500 µg/mL or 25 mg/kg [113,154].

In MRI, SPIONs work as negative contrast agents and can be localized and identified by the signal loss leading to darker areas in the image. Today, SPIONs are used for liver imaging, as they are rapidly engulfed by monocytes and transported to the liver, although the medical community is still reluctant about using SPIONs for other MRI applications, because of the possible toxicity, the inefficient methodology of use, and inconsistent results [94]. The potential toxicity could be put to a good use, though. Hybrid Fe_3_O_4_/Gd_2_O_3_ loaded with cisplatin and tagged with lactoferrin and RGD (a cell endocytosis small peptide) was proved in vitro and in vivo to cross the blood–brain barrier and target brain tumors. By Fenton reaction of the iron ions in the cytoplasm, they induced apoptosis due to iron overload. This process, named ferroptosis, was shown to reduce brain tumors in mice [135] and the number breast cancer cells [155], and also to eliminate the ability of cancer cells to develop resistance to chemotherapy. When studying stem cells or progenitor cells labeled with SPIONs, some authors state that the labeling has no effect on the ability of the cells to differentiate, but other authors claim that this ability is impaired by the iron oxide nanoparticles labeling [44,47,132,156]. Therefore, the cytotoxic reaction of the target cells to SPIONs still requires further examination.

Cationic ethylamine-SPIONs of ultrasmall dimensions showed improved relaxivity values (at smaller dosage) compared to some of the commercial iron oxides, good renal clearance and no toxicity to rats [157]. For enhanced qualities, SPIONs were formulated in various combinations with other metals. Fazal et al. [64] designed a 100 nm iron–gold core–shell SPION complex that is able to give good contrast in MRI and to generate heat when activated by radio frequency fields (a property of nanometer-sized gold particles), in order to destroy the tumor tissue in which it accumulated. This core–shell complex showed no toxicity in vitro and in vivo, but when placed in a radio frequency field it generated high temperatures, and thus, tumor tissue damage. New colloidally stable multi-core iron oxide magnetic nanoparticles, without doping or doped with rare earth metals, were designed in our labs for the use in MRI. Our studies showed that their effects on cells depend on the cell type, cluster design and concentration [158–159]. Asgari et al. [160] produced 50 nm SPION–carbon dot nanoparticles, which were designed for MRI and fluorescence imaging with good cytocompatibility. Park et al. [161] synthesized SPIONs coated with folate containing ^64^Cu for positronic emission tomography and MRI. Cai et al. [162] obtained 12 nm SPIONs coated with a near-infrared fluorescent dye for dual in vivo imagistics (MRI and fluorescence) of Alzheimer’s disease Aβ plaque accumulations. They observed that the nanoparticles are not only appropriate for imaging but they also prevent plaque accumulation and break the already formed aggregates.

Feng et al. [163] developed a SPION sensor coupled with a synthetic protein that recognizes cancerous cells. Upon contact the protein is enzymatically cleaved and eliminated through urine. Followed by ELISA analysis of urine for this protein, the method stands as a useful screening method for tumors. Today, SPIONs are increasingly used for targeting. Hence, it became important to quantify the amount of iron oxides engulfed by the cells. For this, Deda et al. designed a new and improved colorimetric quantification method to measure the concentration of ultrasmall SPIONs (ca. 6 nm) in cell lysates down to tens of nanograms (76 ng/mL Fe or 1.36 μM). This new method added to the qualitative and semi-quantitative methods for detecting and measuring iron content stands as proof for the use of SPIONs in biomedical purposes [164].

### In vitro to in vivo SPIONs effects. Which conclusions should we trust?

Different authors, using different experimental settings, came up with different results and conclusions. Which is the best coating for cellular uptake or for reduced cell uptake and longer circulation time? Or which synthesis conditions are the best for low cytotoxicity? Similar in vitro conditions led to different results, and in vivo analyses changed the entire setting, having no correspondence to the previous in vitro tests. There are very few papers that report the effects of the same type of nanoparticles in vitro and, at the same time, in vivo. Hence, the scientific community stresses the need for both in vitro and in vivo reports on SPION platforms.

To give a few examples, human monocytes were incubated in vitro with CD11b-SPIONs and had an increased upload of nanoparticles due to the specific targeting. However, in the in vivo experiments there was no difference in upload between targeted SPIONs and the control [165]. Szalay et al. [166] worked to determine some correlation of toxicological effects of SPIONs between in vitro and in vivo experiments. They found general toxicity and organ toxicity (lungs) in rats in vivo, but only moderate cell toxicity in vitro (monkey kidney cells). Mahmoudi et al. [43] proved that the cytotoxicity of SPIONs depends also on the cell type. The group worked on three cell types, i.e., heart, kidney, and brain cell lines, with bare SPIONs and SPIONs with negative or positive surface charge. They showed that positively charged nanoparticles are highly toxic and that each cell type responds differently to every type of nanoparticles, genetically, structurally, and metabolically. Uchiyama et al. studied the toxicity of ultrasmall (7 nm) cationic iron oxide particles. They found that at a dose of 10 mg/kg, the ethylamine-coated particles did not induce hemorrhage, clots, inflammation or biochemical imbalance in rats in vivo and ex vivo experiments [157]. This strengthens the point that for every type of nanoparticles, full toxicology analyses with meaningful and adequate in vitro to in vivo correlations are required.

Freund and co-workers [45] used ^56^Fe SPIONs covered in ^14^C oleic acid to radiolabel and quantify the accumulated Fe. They proved that the oleic acid layer is rapidly removed from the iron core and that, although with a small blood circulation time of only 15 min, ^56^Fe SPIONs were not found to be toxic in liver and spleen, where they readily accumulated. This is in contrast with many in vitro studies, in which uncoated SPIONs were shown to be highly toxic [43]. Using Wistar rats, Schlachter et al. [167] tested the release and toxicity of a subcutaneously implanted iron patch made from 15 nm iron oxide particles covered with albumin. Their results also showed that for a period of 6 months there were no toxic effects and no iron overload in any of the organs studied, even though an equivalent of 15 µg/day was released from the mass of the iron patch.

Feng et al. [168] produced a multifunctional nanoplatform consisting of superparamagnetic iron oxide nanospheres coated with ultrasmall up-conversion nanoparticles on mesoporous graphitic-phase carbon nitride and coated with polyethylene glycol for MRI and fluorescence imaging and for photodynamic therapy. This type of nanoplatform seems to be a good one-for-all solution, as it could be controlled to be non-toxic or highly toxic from the outside. In vitro and in vivo analyses reported no cytotoxicity, when not stimulated, and 15% cell viability in vitro and tumor reduction in vivo when activated by a laser.

Horák and collaborators [169] used the toxicity of free Fe ions for antitumor activity. The authors synthesized maghemite nanoparticles covered with poly(*N*,*N*-dimethylacrylamide) that combined with tocopherol acetate would lead to the release of Fe ions from the nanoparticle core with high antitumor activity. They showed that their treatment combination produces Fe ions and lipid peroxidation in vitro. However, in vivo the tumor reduction was determined by the Fe ion release and not by the lipid peroxidation.

Lee et al. [123] synthesized SPIONs functionalized with dopamine and lactobionic acid for MRI and nuclear imaging of the liver. They showed that their formulation targets hepatic cells in vitro and is endocytosed within two hours. In vivo, SPIONs were mainly accumulated in the liver within minutes and, to a smaller extent in spleen, lung, bladder, kidney, making their formulation a good candidate for liver MRI analyses. SPIONs, independent of coating and external magnetic field, seem to accumulate mostly in liver, spleen, and lung [39].

SPIONs were found to be metabolized and incorporated into hemoglobin during the first 1–2 days after injection, with a peak ranging from 5 to 40 days after injection [170]. Iron becomes toxic only when the liver iron concentration exceeds 4 g Fe/kg wet weight [37], while the normal content of iron in the rat liver is 123 μg Fe/g wet weight [127]. In an extensive toxicology experiment on iron nanoparticles, Volkovova et al. [171] found that the lethal dose for 50% of rats is 36 mg Fe/kg body weight. Their study was focused on the SPION effects on the liver, where they found only mild necrosis and lipidosis, but the paper did not suggest any reason for the death of the rats. The routine dose for MRI in rodents ranges between 1 and 20 mg Fe/kg of body weight [172]. These facts are important because it was reported that cirrhosis and liver cancer can form and progress if the liver iron concentration is more than 4 mg/g wet weight [127,173–174]. For humans, the normal dose used for MRI varies between 2.6 mg/kg body weight [127] and 40 mg Fe/kg body weight (Resovist) [81].

The first successful clinical trial employing SPIONs was carried out by Lübbe and collaborators [175]. Since then, many iron oxide nanoparticles were made available commercially and used for MRI analyses on human patients, i.e., Feridex/Endorem, Resovist/Ferucarbotran, Supravist [45,81,156], Ferumoxsil/Lumirem/Gastromark, Feraheme/Ferumoxytol [132,176], Combidex/Sinerem, Clariscan [44]. These formulations have not been approved for use in all countries, and some of them were retracted from the market after unsuccessful reports or due to inefficiency in MRI scanning (ferumoxytol/Feraheme/Rienso, Sinerem/Combidex/ferumoxtran-10, Clariscan) [44,177–179]. Ferumoxtran-10, a formulation of dextran-coated ultrasmall SPIONs, was found to induce side effects such as urticaria, diarrhea, and nausea, all of which were mild and short in duration [73–74]. Larger SPIONs (80–150 nm) are rapidly (10 min) taken up by the reticuloendothelial system and can thus be used for liver imaging. Smaller ones (5–60 nm) are used for imaging of other organs, due to their longer blood circulation time (up to 24 h). These nanoparticles are covered with dextran, carbodextran and/or silica [176]. If also conjugated with specific molecules they can be used for studies of inflammation, heart attack, angiogenesis, apoptosis, gene expression, amyloid plaques, and cancer, using MRI [176].

SPION formulations are used in routine medical applications, despite research that indicates their harmful effects [180]. Chen et al. [156] found that Ferucarbotran/Resovist had inhibitory effects on the osteogenic differentiation of mesenchymal cells and promoted cell migration and the expression of cell cycle and cancer signaling proteins, such as beta-catenin, SSX, and matrix metalloproteinase-2. These effects were noted at concentrations ranging from 50 to 400 µg/mL. Other reports suggested that Feridex does not interfere with the ability of mesenchymal cells to maintain stem status or to differentiate [181]. Also, at concentrations smaller than 300 µg/mL, Ferumoxide/Feraheme is suitable for cell labeling and MRI imaging without affecting chondrogenic differentiation or myocytes in vivo [132–133]. Kostura et al. [182] observed that Feridex-labeled mesenchymal cells were able to differentiate to chondrocytes but not to the osteogenic or the adipogenic line.

It seems that the best way to use SPIONs for medical purposes is via specific design. Ahmadi and collaborators [183] produced a dextran-coated iron oxide nanoworm targeted for pancreatic cells. Their nanosystem was proved to be effective for MRI and fluorescence imaging of specific cancer cells in the pancreas in vitro and in vivo. Also, for pancreatic cancer treatment, Malekigorji et al. [184] developed 50 nm iron–gold core–shell SPIONs with a high quantity of bisnaphthalamido-drug loading. They showed a 12-fold treatment effect in vitro and a 5-fold effect in vivo by laser-heating drug release. In a different approach, Korpi et al. [185] used electroporation and rotating incubation to label bone marrow mononuclear cells to detect acute myocardial infarction areas in vitro and in vivo. For controlled drug release, 12 nm SPIONs functionalized with poloxamer and heparin and loaded with doxorubicin, were used for 120 h of slow drug release against HeLa cells in vitro [38]. For the treatment of resistant glioma cells, Luque-Michel et al. [186] obtained spherical polymer platforms made of poly(lactic-*co*-glycolic acid) containing SPIONs and doxorubicin, which were covered with surfactants. They proved the effectiveness of these platforms in crossing the blood–brain barrier and in MRI of tumors. In addition, the platforms showed cytotoxic effects on glioma cells and neuronal stem cells obtained from patients. SPIONs show promise in dentistry as well. Calcium phosphate composites combined with iron–gold core–shells SPIONs conjugated with bone morphogenetic protein were showed to have a good dental pulp and dentin treatment effect and also give contrast for dental MRI for up to seven weeks [187].

## Conclusion

From the many studies involving SPIONs, here are a few general indications regarding their characteristics and effects:

1) The shape of SPIONs can determine how cells react to them. Spherical iron oxide particles are easier to produce and, therefore, many studies refer to them. Cubic SPIONs were shown to have superior magnetization properties, while octopod SPIONs were proved to give a better MRI signal quality than spherical particles.

2) Naked SPIONs are toxic for cells in vitro, while their magnetization properties are preserved. In vivo, naked SPIONs are rapidly cleared from blood circulation; at concentration above 40 mg/kg they become toxic. If nanoparticles are previously covered with an organic material, which is later stripped from the particles in vivo, the toxicity is reduced significantly.

3) Polymer-coated SPIONs give better biocompatibility in vitro and are better endocytosed, but researchers still argue about the impaired magnetization properties of coated SPIONs.

4) Among coated SPIONs, dextran-coated SPIONs are better endocytosed by tumor cells and tend to accumulate at tumor sites. Also, they have a small circulation time and were shown to cross the blood–brain barrier. PEG-coated SPIONs have a longer blood circulation time and also accumulate at tumor sites. PVA-coated SPIONs are the most colloidally stable and therefore most commonly used particles. They are approved for MRI in patients. Other coatings such as Si or polyethyleneimine were shown to yield a very long circulation time. Au-coated SPIONs are resistant to clearance, in contrast to oleic acid-coated SPIONs, which are cleared within minutes.

5) Zeta potential or electrostatic charge of the SPIONs surface determine the way nanoparticles will be treated by the cells. A positive potential of 0–25 mV will make the SPIONs adhere electrostatically to cell membranes and was shown to be toxic in utero. Particles with a negative potential close to zero are accumulated in tissues and are rapidly recognized by the Kupffer macrophages in the liver. The higher the zeta potential, the less toxic the particles are to cells.

6) SPIONs of intermediate size (20–50 nm) have a longer circulation time (days) and were considered suitable in the majority of studies with the best endocytosis rate. SPIONs larger than 80 nm have ferromagnetic properties and those larger than 100 nm can only reach a minority of tissues. SPIONs smaller than 25 nm have superparamagnetic properties. The MRI-approved dimensions are between 5 and 15 nm and are also suitable for hyperthermia. Particles smaller than 10 nm can be dangerous when reaching the nucleus, as they are, too, easily distributed in vivo. Very large (above 90 nm) or very small (below 20 nm) SPIONs were found to either be taken up by the reticuloendothelial system within minutes or to have very long circulation times.

7) Once endocytosed, SPIONs can be found in lysosomes and also free in the cytoplasm, where they can be degraded in a safe way by the cells. Also, they can induce cell death via the ferroptosis cascade. This depends on the cell type and can also depend on concentration/dose. Monocytes and macrophages eventually phagocytose SPIONs from blood, but opsonization of SPIONs might not be important for this process. All types of SPIONs ultimately accumulate in vivo in liver and spleen. Endocytosis can inhibit heat transfer in hyperthermia or can provide a cell selectivity of the process, only harming the targeted cells.

8) The efficacy of SPIONs in hyperthermia treatment depends on the size, as long as the SPIONs exhibit only a single magnetic domain. It also depends on the viscosity of the tumor site because viscous material drastically reduces the heat transfer. Hyperthermia can induce cell death through increasing the local temperature up to 41–45 °C, but also simply through the applied magnetic field without significant temperature increase. Core–shell particles consisting of SPIONs and other metals (Zn, Mn, Ni) can have increased hyperthermia efficacy.

9) Recently, SPIONs were observed to be efficient in photothermal therapy under near-infrared laser irradiation. MRI applicability is studied extensively and many formulations were already approved for patient use. They are especially used for MRI of liver and spleen, where they naturally accumulate. Combined synthesis with Au, Cu, carbon dots, or rare metals improves the MRI properties of SPIONs.

With this review we want to emphasize the fact that it is crucial to gain more insight into the in vivo toxicology effects (short term and long term) in order to truly understand which types of nanoparticles are safe. In vitro analyses are still needed, of course, to test hypotheses and prove in a safe and controlled way that certain nanoparticles and nanoplatforms are biocompatible. But it is our belief, that we have reached a point where knowledge about biocompatibility alone is not sufficient any more. Also, in vitro analyses are limited in time scale and spatial dimensionality, and unidirectional (restricted to single cell types). Co-cultures and tridimensional in vitro analyses of nanoparticles might give more insight, but they are also limited. The possibility of organ-on-a-chip constructs may be a solution for the examination of organ-directed effects of nanoparticles in order to avoid the in vivo exposure of lab animals. Specific biomedical application of SPIONs might be available in the near future, but we still need more insight into their mechanisms of action.
